# Kinetic characteristics of Wushu Changbing PiJi: decoupling sex-specific adaptive strategies via functional principal component analysis

**DOI:** 10.3389/fspor.2026.1815388

**Published:** 2026-05-20

**Authors:** Pengfei Zheng, Yupeng Shen, Yue Sun, Shuxin Wang, Yiyi Qiu, Jiewei Ma, Huahong Sun, Lianzhen Ma

**Affiliations:** 1 School of Physical Education and Sports Science, South China Normal University, Guangzhou, China; 2 School of Physical Education and Health, Guangdong Polytechnic Normal University, Guangzhou, China

**Keywords:** Wushu Changbing, PiJi, three-dimensional ground reaction force, multivariate functional principal component analysis, sex differences, kinetic strategy

## Abstract

**Background:**

Wushu Changbing PiJi is a representative striking action in Wushu long-weapon routines, with its most iconic form derived from the Ma's Tongbei Wushu school, whose staff techniques represent a direct lineage of the Ming Dynasty Shaolin staff system. Biomechanically, it is a high-velocity, long-lever striking action analogous to other open-chain striking tasks. However, sex-specific ground reaction force (GRF) control and force transmission strategies during this movement remain unclear.

**Objective:**

This study aimed to characterize sex-specific kinetic adaptations during Wushu Changbing PiJi using multivariate functional principal component analysis (mfPCA) of three-dimensional ground reaction forces (3D GRFs).

**Methods:**

Fifty elite collegiate Wushu Changbing athletes performed maximal-effort PiJi trials with synchronized optical motion capture and dual force plates. mfPCA was used to extract the core spatiotemporal modes of normalized 3D GRFs. Linear mixed-effects models were then applied to test sex effects on five principal component scores and 16 discrete kinetic metrics.

**Results:**

No significant sex differences were observed in PC1, PC2, or PC5, indicating a shared core spatiotemporal kinetic structure across sexes. In contrast, significant sex effects were found in PC3 and PC4, reflecting differences in fine-grained control strategies.Males demonstrated greater total peak implement kinetic energy, alongside increased peak lateral force, mean propulsive force, and lateral force standard deviation, consistent with a propulsion-dominant but less stable kinetic profile. Females showed higher PC3 and vertical impulse, suggesting a control-oriented braking and stabilization strategy.

**Conclusion:**

Wushu Changbing PiJi appears to be governed by a shared core force-generation rhythm, while sex-specific adaptations are primarily expressed through a propulsion-stability trade-off. These findings support sex-specific training interventions that preserve the common temporal template while targeting stability control in males and power-conversion capacity in females.

## 1 Background

The manipulation of long-handled implements represents a quintessential paradigm of functional limb extension in human tool use, evolving alongside the progression of civilization. Within the dual contexts of historical self-defense necessities and modern competitive combat, the PiJi technique—a representative striking action in Wushu long-weapon routines, with its most iconic form derived from the Ma's Tongbei Wushu school, whose staff techniques represent a direct lineage of the Ming Dynasty Shaolin staff system (1, 2)—has evolved into a core long-range scoring maneuver in modern competitions. From a biomechanical and motor control perspective, this technique shares kinematic similarities with other two-handed weapons (e.g., Historical European Martial Arts, Kendo, and Southeast Asian long staffs), reflecting universal principles of human implement manipulation for extended striking.

Biomechanically defined, Wushu Changbing PiJi is a high-velocity, open-kinetic-chain movement involving a long lever arm (3). This pattern shares biomechanical homology with the golf swing, baseball batting, and tennis serving, providing a robust framework for elucidating underlying mechanical transmission (4). In the study of high-velocity swinging kinetics, Ground Reaction Forces (GRF)-dominated by lower limb drive-have been established as the initiating link in energy generation and transfer (5, 6). Both the magnitude and vector characteristics of GRF strongly influence the kinematic performance of the end-effector (7–9). Research on swinging motions indicates that Peak vertical and posterior GRF of the lead leg positively correlate with swing velocity and drive momentum transmission upward along the kinetic chain (9–11). Beyond force magnitude, anticipatory Center of Pressure (COP) shifts and trunk rotation kinetics further facilitate the momentum transfer model from the ground to the distal end (12–14).

However, maximizing force is not the exclusive pathway to enhanced performance; precise temporal coordination of force generation plays an equally pivotal role (15). Research indicates that multi-dimensional force synchrony may outweigh unidimensional explosive magnitude, as seen in tennis serving and rapid base-running transitions in baseball, which rely heavily on minimizing time to peak force rather than solely on absolute magnitude (16, 17). Neurophysiologically, this precise spatiotemporal regulation reflects the superior intermuscular coordination and efficiency characteristic of elite athletes (18, 19). Applying Screw Theory (20), revealed that elite practitioners exhibit an “invariant pitch,” demonstrating highly consistent temporal coupling.Viewed through the lens of Generalized Motor Program (GMP) theory and skill acquisition principles, this conserved spatiotemporal sequencing likely reflects an invariant feature of elite motor skills, suggesting a shared underlying motor template across populations even as execution parameters (e.g., absolute force) vary (21, 22). While foundational lower-limb strength underpins these high-velocity movements (23, 24), targeted loading interventions further verify the plasticity and training sensitivity of these spatiotemporal kinetic parameters (25–27).

Current literature elucidates that significant sex-specific kinetic heterogeneity exists in the manipulation of long lever-arm implements (28–31). Empirical evidence from homologous swinging sports (e.g., golf and tennis) indicates that such heterogeneity often translates into distinct kinetic strategies: males tend to rely more on absolute force generation (propulsion-dominant), whereas elite females frequently optimize pelvic-torso coordination and lead-leg braking (stability-oriented) (29, 30, 32). However, current Wushu Changbing pedagogy often relies on experience-driven paradigms, which may overlook disparities in neuromuscular control arising from physiological heterogeneity (33). The resulting biomechanical mismatch might not only restrict competitive potential but also potentially elevate the risk of sports injury (34, 35). In light of these limitations, this study aimed to employ multi-dimensional biomechanical approaches to systematically investigate the sex-specific adaptive mechanisms inherent in the PiJi technique. The findings are intended to support the development of evidence-based, sex-specific training protocols and advance the scientific progression of Wushu Changbing.Based on the aforementioned background, this study proposes the following hypotheses: (A) Significant sex differences exist in the kinetic control of the Wushu Changbing PiJi technique between male and female athletes. (B) Driven by disparities in physiological structure and neuromuscular control characteristics, male and female athletes adopt distinct kinetic strategies during PiJi. These differences are primarily manifested through a “propulsion-stability” regulatory mechanism characterized by kinetic metrics. (C) Despite sexual dimorphism in force magnitude and control strategies, the Wushu Changbing PiJi technique remains governed by a core kinetic spatiotemporal structure shared across sexes.

## 2 Materials and methods

### 2.1 Participants

An equivalent power analysis approximating the mixed-model structure via a repeated-measures framework in G Power (ANOVA: Repeated measures, within-between interaction; effect size f=0.25; α=0.05; assumed trial-to-trial correlation r=0.5) confirmed that the 700 total observational samples (50 participants × 14 trials) yielded an actual statistical power (1−β=0.80) exceeding 0.95, ensuring highly adequate statistical power for the linear mixed-effects modeling. The cohort consisted of 30 males (age: 22.1 ± 1.5 years; height: 176.8 ± 6.0 cm; body mass: 74.7 ± 9.3 kg) and 20 females (age: 21.9 ± 1.5 years; height: 167.5 ± 7.2 cm; body mass: 62.7 ± 9.3 kg).

Inclusion criteria were strictly defined as follows: (a) Right-hand dominance, to minimize potential confounding effects of handedness on movement patterns; (b) Free from any musculoskeletal injuries within the six months prior to the experiment; and (c) A top-five ranking in their respective divisions and weight classes at the National Collegiate Wushu Championships within the past year. Notably, this tournament comprises 8 divisions (by sex and university classification) and 8 weight classes per division, yielding 64 independent competition units and a baseline pool of 320 top-five athletes annually. Our recruited cohort (*n* = 50) represents approximately 15.6% of this annual pool, ensuring strong representativeness and logistical feasibility. Furthermore, all participant rankings were strictly verified using official certificates and tournament results booklets issued by the Chinese Wushu Association.

Prior to data collection, all participants were fully informed of the experimental procedures and provided written informed consent. The study protocol was approved by the Ethics Committee of the School of Physical Education and Sports Science at South China Normal University (Approval No. SCNU-SPT-2025-033) and was conducted in strict accordance with the ethical guidelines of the Declaration of Helsinki.

### 2.2 Experimental protocol

All participants initially underwent a 20 min standardized warm-up protocol comprising jogging, dynamic stretching, and unloaded swings to ensure neuromuscular activation and environmental adaptation. During the formal data collection phase, participants adopted a split-stance posture, with their feet positioned individually at the centers of two adjacent 3D force plates, thereby achieving physical decoupling of unilateral ground reaction force (GRF) signals. Following standardized instructions, participants were required to complete 14 valid PiJi trials with Maximal Voluntary Effort (MVE) (68, 69). To ensure within-participant kinetic consistency, the force-time waveforms of the 14 repeated trials were subjected to rigorous manual inspection and overlay fitting. A trial was classified as valid if it met three specific criteria: (1) correct technical execution throughout a complete movement cycle, as verified by an on-site expert; (2) accurate impact on the predefined target zone (simulating an opponent's head and shoulder vicinity); and (3) complete and uncorrupted acquisition of all experimental data. To mitigate the effects of cumulative fatigue and maintain performance stability, recovery intervals between trials were self-selected by the participants (36).

### 2.3 Movement task and temporal segmentation

**Figure 1:**
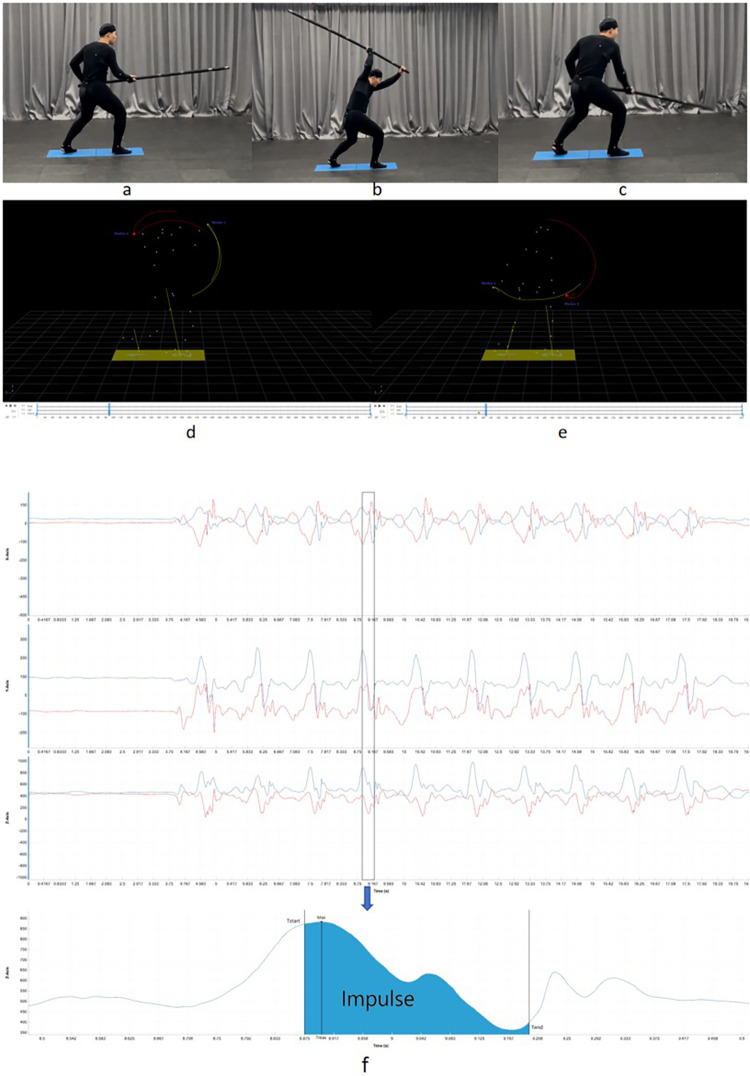
Temporal segmentation and data extraction of the Wushu Changbing PiJi movement. **(a–c)** Sequential illustration of the participant's technical execution: **(a)** Preparatory posture and overhead backswing phase; **(b)** Onset $\left(t_{\mathrm{start}}\right)$: Defined by the peak vertical displacement of the implement, marking the critical kinetic transition from energy storage during the backswing to the acceleration of the downswing; **(c)** Termination $\left(t_{\mathrm{end}}\right)$: The state of kinematic quiescence following the strike. **(d–e)** Temporal determination based on the spatial trajectories of the implement's distal end (Marker 1, yellow line) and reference point (Marker 4, red line): **(d)** $t_{\mathrm{start}}$: Identified as the static peak of the backswing phase, signaling the initiation of downward acceleration; **(e)** $t_{\mathrm{end}}$: Corresponds to the final cessation point of the complete striking trajectory, representing total momentum dissipation and the conclusion of the process. The valid analysis interval covers the period from $t_{\mathrm{start}}$ to $t_{\mathrm{end}}$. **(f)** Extraction of experimental data and key kinetic metrics.

In this study, the Wushu Changbing PiJi, characterized by typical sagittal-plane dominance, served as the testing paradigm (Figure 1). Participants initiated the movement from a split-stance preparatory posture, executing a substantial backswing to store elastic energy through agonist stretching, followed immediately by an explosive vertical downward slash driven by the whole-body kinetic chain. After excluding artifacts using Mokka software (v0.6.2), the valid analysis period was segmented based on the kinematic markers of the implement (Marker 1 and Marker 4). The onset ($t_{\mathrm{start}}$) was defined as the instant of peak vertical displacement during the backswing (Figures 1b,d). At this point, vertical velocity reached zero, marking the kinetic transition from potential energy storage to the acceleration phase. The termination $\left(t_{\mathrm{end}}\right)$ was identified as the kinematic point of quiescence post-impact (Figures 1c,e), representing the moment of complete momentum dissipation and the restoration of relative stability. Consequently, time-series data ranging from $t_{\mathrm{start}}$ to $t_{\mathrm{end}}$ were extracted for statistical analysis.

### 2.4 Data acquisition and processing

**Figure 2:**
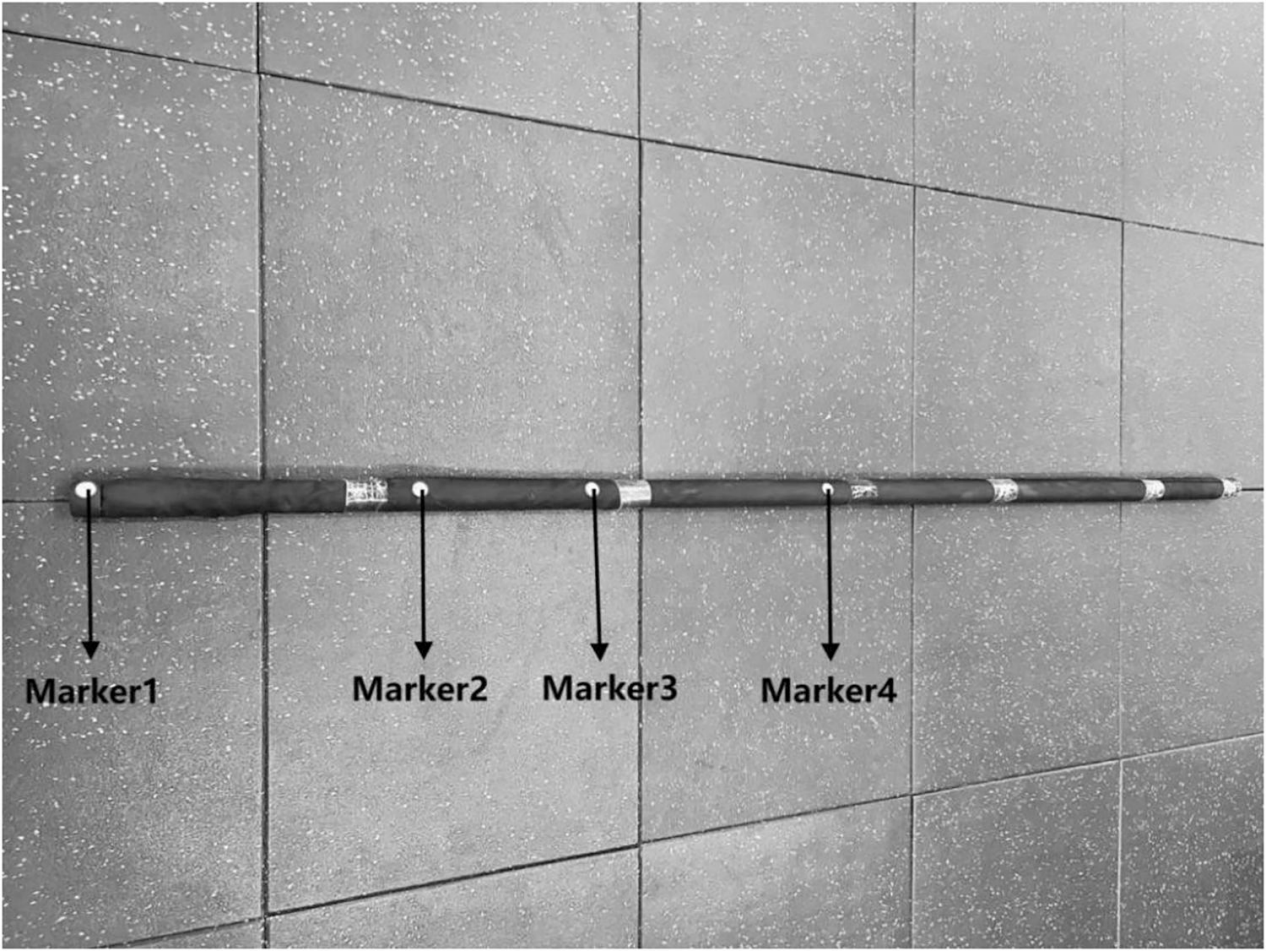
Marker placement configuration on the Wushu Changbing implement.

To ensure synchronized signal acquisition, the study employed a Nokov optical motion capture system (240 Hz; Nokov Science & Technology, Beijing, China) integrated with two Kunwei 3D force plates (1000 Hz; Kunwei Technology, Jiangsu, China). A hardware synchronization unit was utilized to guarantee spatiotemporal consistency between kinematic and kinetic data. Participants held a standard Wushu Changbing implement (length: 2.0 m; mass: 1.0 kg) and adopted a split-stance posture, with each foot positioned on an individual force plate to isolate the acquisition of ground reaction forces (GRF). Reflective markers were affixed along the shaft of the implement (at 0, 37, 61, and 100 cm), and their vertical trajectories served as the temporal reference for defining movement phases (Figure 2).

The data analysis workflow was executed automatically within a Python 3.9 environment. All raw signals underwent synchronous smoothing via a fourth-order zero-phase Butterworth low-pass filter (cut-off frequency: 50 Hz) to eliminate high-frequency noise while preserving dynamic characteristics.

#### 2.4.1 Extraction of kinetic characteristics of 3D ground reaction forces

All functional data processing and multivariate functional principal component analysis (mfPCA) computations were executed in a Python 3.9 environment utilizing the scikit-fda library. To eliminate high-frequency noise interference while ensuring the integrity of the biomechanical features, the raw 3D GRF signals were first smoothed using a fourth-order zero-phase Butterworth low-pass filter with a cutoff frequency of 50 Hz (37). After interpolating to a 101-point time grid, per-curve Z-score standardization was applied independently across the three orthogonal axes. By unitizing variance and eliminating absolute amplitude differences, this step ensures the mfPCA focuses strictly on temporal morphological features. This mathematically mitigates structural skew from magnitude disparities and minimizes potential artifacts introduced by different body mass scaling methods (38).

To capture the cross-dimensional spatiotemporal coupling, the standardized data from the three axes for each trial *i* were concatenated into a joint high-dimensional feature vector $X_{i}$ (Equation 1):

$$X_{i}=[F_{x}(t_{1}),\ldots ,F_{x}(t_{101}),F_{y}(t_{1}),\ldots ,F_{y}(t_{101}),F_{z}(t_{1}),\ldots ,F_{z}(t_{101}){]}^{T}\in R^{303}\;$$ (1)

Based on the Karhunen-Loève expansion theory, eigen-decomposition was performed on the joint covariance operator. Each observed function $X_{i}(t)$ is approximated as a linear combination of the overall mean trajectory μ(t) and orthogonal principal component functions $\phi_{k}(t)$ (Equation 2):

$$X_{i}(t)\approx \mu (t)+\overset{K}{\underset{k=1}{\sum }}\xi_{ik}\phi_{k}(t)\;$$ (2)

where $\xi_{ik}$ represents the principal component score quantifying the individual's deviation along the *k* -th mode of variation.

**Figure 3:**
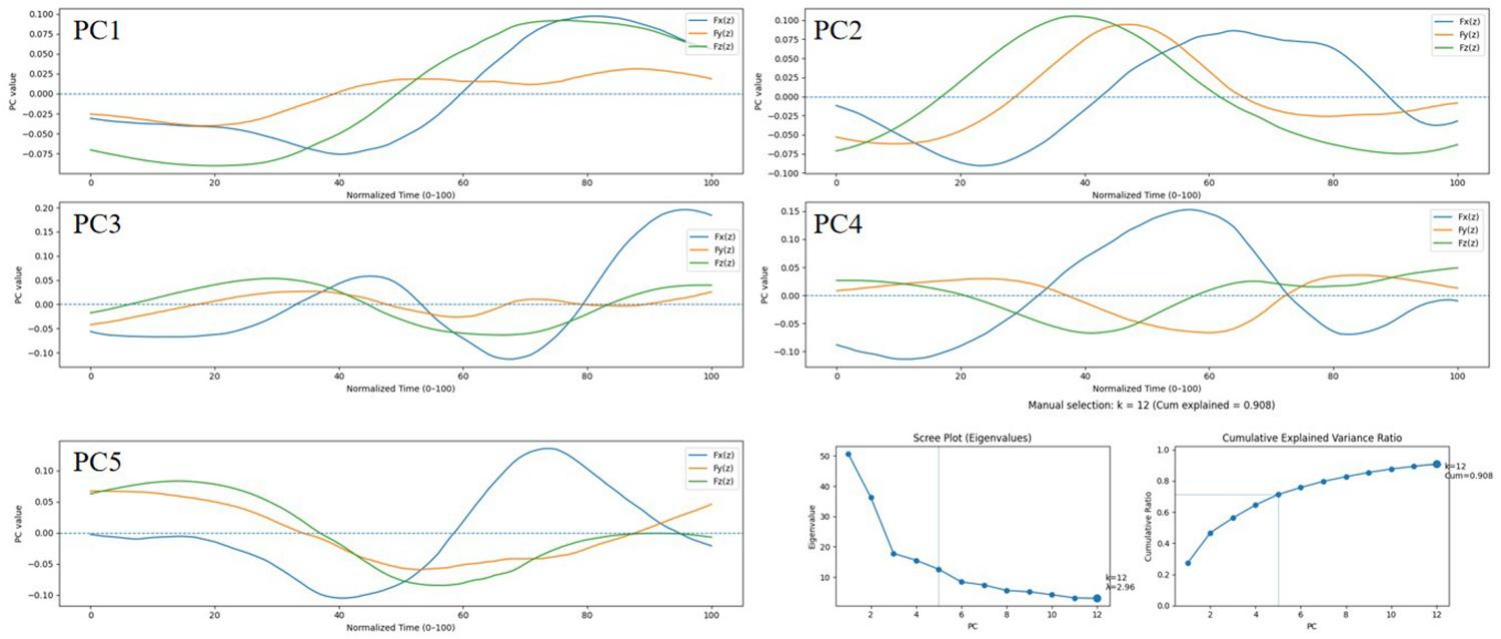
Five principal kinetic patterns. PC1-PC5 display the first five functional principal components (fPCs) characterizing the spatiotemporal features of 3D Ground Reaction Forces (3D GRF). The waveform curves correspond to the Medial-Lateral (Fx, blue), Anterior-Posterior (Fy, orange), and Vertical (Fz, green) components, respectively. The scree plot and cumulative variance ratio confirm that the retention of the first five principal components (accounting for 71.2% of the cumulative variance) is sufficient to constitute a robust low-dimensional orthogonal basis.Physically, the late-phase Fx spike and concurrent Fy and Fz attenuation in PC3 characterize terminal braking and stabilization, whereas the mid-phase coupled fluctuations of Fx and Fy in PC4 denote the dynamic lateral stability-propulsion trade-off.

As illustrated in Figure 3, based on the determination of the inflection point in the Scree Plot, the first five principal components (PC1-PC5) were retained, cumulatively explaining 71.2% of the total system variance. While a 90% threshold is occasionally utilized in low-dimensional contexts, established statistical guidelines emphasize that for high-dimensional data (e.g., p=303), an optimal cut-off typically falls between 70% and 90% (39). Retaining additional components to artificially reach a 90% threshold in dense kinematic datasets frequently leads to the over-extraction of high-frequency noise and non-functional biomechanical artifacts (40, 41). Empirically, variance thresholds near 70% are routinely adopted and validated as sufficient for isolating core structures in complex human and health-related datasets (42, 43). Given the physical interpretability of these five mfPCA principal components, they were adopted as key metrics for subsequent modeling (44). While inherently data-driven mathematical constructs rather than neurophysiological synergies (45), these principal components effectively capture macroscopic, coordinated force-application features (46–48). Consequently, they are termed “principal kinetic patterns (49)” herein.

#### 2.4.2 Calculation of the total kinetic energy of the changbing implement

To address the challenges associated with implement deformation during high-speed motion and the spatial limitations of the motion capture volume, this study employed a “Virtual Marker Reconstruction” technique. The Changbing was modeled as a homogeneous slender rigid body. Based on markers positioned on the mid-to-posterior sections of the shaft, a rigid body model was established to compute the distal trajectory and total kinetic energy via the following steps:

##### 2.4.2.1 Trajectory reconstruction and rigid body transformation

Initially, a reference template, denoted as $p_{\mathrm{ref}}$, was established based on a static calibration trial. During the dynamic trials, the Kabsch algorithm was employed to compute the optimal rotation matrix R(t) and translation vector T(t) for each frame. The rigid body pose P(t) was determined by minimizing the Root Mean Square (RMS) error (Equation 3):

$$\underset{R,T}{\min}\overset{4}{\underset{i=1}{\sum }}\|(R\cdot p_{\mathrm{ref},i}+T)-p_{\mathrm{obs},i}(t){\|}^{2}$$ (3)

Based on the computed results, the instantaneous spatial coordinates of the implement tip, denoted as $P_{\mathrm{tip}}(t)$, were derived (Equation 4):

$$P_{\mathrm{tip}}(t)=R(t)\cdot p_{\mathrm{ref},\mathrm{tip}}+T(t)\;$$ (4)

##### 2.4.2.2 Velocity calculation

A zero-phase low-pass filter with a cut-off frequency of 12 Hz was applied to the reconstructed coordinate data to eliminate noise. Subsequently, linear and angular velocities were computed using the central difference method (Equations 5 and 6):

$$v(t)=\frac{r(t+\Delta t)-r(t-\Delta t)}{2\Delta t}$$ (5)

where Δt represents the sampling interval, and r denotes the position vector.

$$\omega (t)=\frac{u(t)\times \dot{u}(t)}{\|u(t){\|}^{2}}$$ (6)

where u(t) denotes the vector aligned with the longitudinal axis of the shaft.

##### 2.4.2.3 Calculation of total kinetic energy

According to König's theorem, the total kinetic energy of the Changbing implement, denoted as $E_{k}(t)$, comprises two components: the translational kinetic energy of its center of mass (COM) and the rotational kinetic energy about the COM. It is calculated using the following Equation 7:

$$E_{k}(t)=\frac{1}{2}m\|v_{\mathrm{cm}}(t){\|}^{2}+\frac{1}{2}I_{\mathrm{cm}}\|\omega (t){\|}^{2\;}$$ (7)

where $v_{\mathrm{cm}}(t)$ represents the linear velocity of the shaft's center of mass, and $I_{\mathrm{cm}}$ denotes the moment of inertia of the slender rod about its center of mass

#### 2.4.3 Definition and calculation of kinetic metrics

In this study, the acquired ground reaction force (GRF) signals were processed to compute core metrics across four dimensions. Herein, the subscript i denotes the three orthogonal directions: X(Lateral),Y(Propulsive), and Z(Vertical). The calculation methodologies and physical implications of each metric are elucidated as follows:

1. Impulse: Impulse(X), Impulse(Y), and Impulse(Z) characterize the change in momentum in the lateral, propulsive, and vertical directions, respectively. This metric is obtained by integrating the force-time curve, reflecting the cumulative effect of the force exerted during a specific movement cycle (Equation 8):
  $$\mathrm{Impulse}(i)=\overset{t_{\mathrm{end}}}{\underset{t_{\mathrm{start}}}{\int }}F_{i}(t)\;\mathrm{dt}$$ (8)
  where $F_{i}(t)$ represents the instantaneous force value in direction i at time t, and the integration interval from $t_{\mathrm{start}}$ to $t_{\mathrm{end}}$ encompasses the entire valid movement analysis period.
2. Peak Force (Max): Max(X), Max(Y), and Max(Z) are defined as the maximum instantaneous loads achieved in each respective direction during the explosive phase of the movement. These metrics are utilized to quantify the maximal intensity and explosive power of the action (Equation 9):
  $$\mathrm{Max}(i)=\max(F_{i}(t))\;\mathrm{for}\;t\in [t_{\mathrm{start}},t_{\mathrm{end}}]$$ (9)
3. Mean Force (Mean): Mean(X), Mean(Y), and Mean(Z) represent the average force output in their respective directions. These metrics are utilized to evaluate the sustained load level throughout the entire movement duration. They are calculated using the arithmetic mean (Equation 10):
  $$\mathrm{Mean}(i)=\frac{1}{N}\overset{N}{\underset{n=1}{\sum }}F_{i}(t_{n})\;$$ (10)
  where N denotes the total number of sampled data points within the analysis window, and $F_{i}(t_{n})$ represents the force value at the n-th frame.

### 2.5 Statistical analysis

All statistical procedures were performed using JASP software (Version 0.95.4.0). To eliminate the confounding effects of anthropometric differences, all kinetic parameters were normalized to body mass prior to model fitting. Linear Mixed-Effects Models (LMMs) were employed to investigate the effect of sex on the 3D kinetic metrics. The model structure defined sex as a fixed effect, while participants were treated as a random effect with random intercepts to account for the inter-subject variability and the nested structure of multiple observational trials. and inter-subject variability. The significance of the fixed effect was determined via Type III Analysis of Variance (ANOVA) based on Satterthwaite's approximation for degrees of freedom, which robustly accounts for the unequal group sizes (30 males vs. 20 females). All statistical tests were two-tailed, and the level of significance was set at α=0.05. To strictly control the Type I error rate inflation arising from multiple hypothesis testing across the 21 dependent variables (5 principal components and 16 discrete metrics), all resulting p-values were adjusted using the Benjamini-Hochberg False Discovery Rate (FDR) procedure. Furthermore, to evaluate the practical magnitude of the observed sex differences, effect sizes were calculated using Cohen's d, with thresholds interpreted as small (≥0.2), moderate (≥0.5), and large (≥0.8).

## 3 Results

### 3.1 Sex differences in the principal components of 3D ground reaction forces

**Figure 4:**
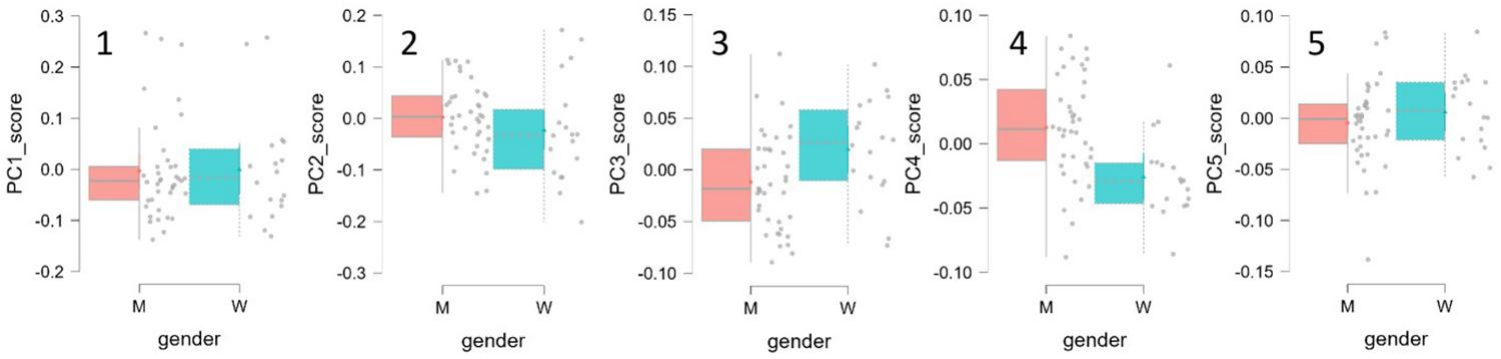
Box plots of sex distributions for principal component scores (PC1-PC5). The horizontal axis represents sex, where M(Men) indicates males and W(Women) indicates females; the vertical axis represents the principal component(PC) scores, with 1-5 denoting PC1-PC5, respectively. The box plots display the median (red line), interquartile range(box), and outliers (scattered points) for each group.

Based on Multivariate Functional Principal Component Analysis (mfPCA), this study established five core principal components governing the Wushu Changbing PiJi technique (47, 49–51) (Figure 3 and Table 1). Among these, PC1 and PC2 precisely quantified the magnitude and temporal coupling characteristics of overall explosive power. Meanwhile, PC3 through PC5 profoundly decoded micro-regulatory kinetic strategies, including active braking mechanisms, the lateral stability-propulsion trade-off, and vertical drive vector synthesis. The results (Figure 4, Table 2) indicated no statistically significant main effect of sex on PC1[F(1, 54.92) = 0.009, *p* = 0.926], PC2[F(1, 54.91) = 1.191, *p* = 0.280], or PC5[F(1, 54.47) = 0.868, *p* = 0.356]. In contrast, the main effect of sex was significant for PC3[F(1, 54.99) = 5.439, *p* = 0.023], with females scoring significantly higher than males. Similarly, a significant sex difference was observed for PC4[F(1, 54.81) = 12.28, *p* < 0.001], characterized by males scoring significantly higher than females.

**Table 1:** Eigenvalues and variance explained by the first five functional principal components (fPCs).

PCs	Eigenvalue	Explained_ratio	Cumulative_ratio
PC1	50.72	27.18%	27.18%
PC2	36.28	19.44%	46.61%
PC3	17.81	9.54%	56.16%
PC4	15.52	8.31%	64.47%
PC5	12.57	6.73%	71.20%

**Table 2:** Statistical results of sex differences across the first five functional principal components.

	Male		Female						
PCs	Estimate	SE	Estimate	SE	df	t	p	Cohen's d	VS-MPR*
PC1	−0.003	0.016	−4.617 × 10^−4^	0.024	54.92	−0.093	0.926	−0.025	1
PC2	0.002	0.013	−0.023	0.019	54.91	1.091	0.28	0.294	1.032
PC3**	−0.012	0.008	0.02	0.011	54.99	−2.332	0.023	−0.629	4.189
PC4***	0.013	0.006	−0.026	0.009	54.81	3.505	<.001	0.947	57.221
PC5	−0.005	0.007	0.006	0.01	54.47	−0.932	0.356	−0.253	1.001

* *p* < 0.05, ** *p* < 0.25, *** *p* < 0.001.

### 3.2 Sex differences in 3D kinetic metrics

**Figure 5:**
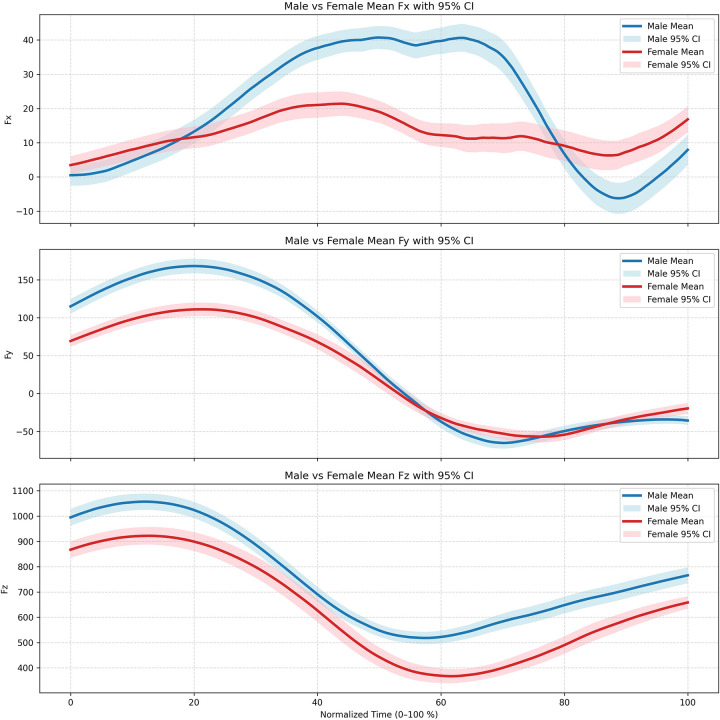
Sex-separated mean curves of 3D ground reaction forces with 95% confidence intervals. The *X*-axis represents the normalized movement cycle (0%–100%), encompassing the entire process from the initiation to the completion of the strike. The *Y*-axis corresponds to the three-dimensional ground reaction force components: Fx, Fy, and Fz.Solid lines denote the group means (males in blue, females in red), and the shaded bands represent the 95% confidence intervals.

The sex-separated mean curves of the 3D ground reaction forces (3D GRF) with 95% confidence intervals throughout the entire PiJi movement cycle are illustrated in Figure 5.

The results (Table 3) indicated no statistically significant main effect of sex on Impulse(X) [F(1,54.59) = 0.678, *p* = 0.414], Impulse(Y) [F(1,55.03) = 3.745, *p* = .058], SD(Z) [F(1,54.85) = 0.295, *p* = 0.589], SD(Y) [F(1,54.71) = 1.673, *p* = 0.201], Time_at_Max (Z)[F(1,54.91) = 0.74, *p* = 0.393], Time_at_Max(X) [F(1,54.91) = 0.863, *p* = 0.357], and Time_at_Max(Y) [F(1,54.91) = 0.767, *p* = 0.385]. In contrast, a significant main effect of sex was observed for Impulse(Z) [F(1,54.89) = 9.629, *p* = 0.003], with females exhibiting significantly higher values than males. A highly significant sex difference was present in the total peak kinetic energy, T_full_peak_J[F(1,55.25) = 6.615, *p* = 0.013], where males showed significantly higher values than females. Furthermore, males demonstrated significantly higher values than females in kinetic metrics across the X and Y directions, including Max(X) [F(1,54.20) = 6.797, *p* = 0.012], Mean(X) [F(1,54.06) = 4.146, *p* = 0.047], Mean(Y) [F(1,54.84) = 9.762, *p* = 0.003], and SD(X) [F(1,54.77) = 13.98, *p* < 0.001].

**Table 3:** Statistical results of sex differences in 3D kinetic metrics.

	Male		Female						
3D Kinetic metrics	Estimate	SE	Estimate	SE	df	t	p	Cohen's d	VS-MPR
T_full_peak_J**	1.403	0.047	1.186	0.070	55.25	2.572	0.013	0.692	6.582
Impulse(X)	0.095	0.008	0.083	0.012	54.59	0.823	0.414	0.223	1
Impulse(Y)	0.215	0.016	0.159	0.024	55.03	1.935	0.058	0.522	2.225
Impulse(Z)**	3.521	0.109	4.119	0.159	54.89	−3.103	0.003	−0.838	20.96
Max(X)**	1.125	0.064	0.826	0.095	54.2	2.607	0.012	0.708	7.036
Max(Y)	2.683	0.145	2.229	0.213	54.74	1.763	0.084	0.477	1.774
Max(Z)	17	0.446	16.77	0.655	54.86	0.286	0.776	0.077	1
Mean(X)*	0.274	0.021	0.197	0.031	54.06	2.036	0.047	0.554	2.573
Mean(Y)**	0.63	0.043	0.391	0.063	54.84	3.124	0.003	0.844	22.05
Mean(Z)	10.29	0.131	10.05	0.193	54.56	1.054	0.297	0.285	1.02
SD(X)***	0.485	0.021	0.345	0.031	54.77	3.738	<.001	1.01	107.3
SD(Y)	1.412	0.083	1.22	0.123	54.71	1.294	0.201	0.35	1.14
SD(Z)	4.112	0.244	4.348	0.359	54.85	−0.543	0.589	−0.147	1
Time_at_Max(X)	6.566	0.219	6.928	0.322	54.91	−0.929	0.357	−0.251	1
Time_at_Max(Y)	6.454	0.217	6.79	0.318	54.91	−0.876	0.385	−0.236	1
Time_at_Max(Z)	6.473	0.217	6.804	0.318	54.91	−0.860	0.393	−0.232	1

Estimate represents the estimated coefficient; SE, Standard Error; df, degrees of freedom; VS-MPR, Vovk-Sellke Maximum *p*-Ratio. Max, peak value; Mean, average value; SD,  Standard Deviation (indicating variability); Time_at_Max, time to peak; T_full_peak_J,  total peak kinetic energy.

* *p* < 0.05, ** *p* < 0.01, *** *p* < 0.001.

## 4 Discussion

Utilizing Linear Mixed-Effects Models (LMMs), this study systematically characterized the sex-based differences in the PiJi technique among elite Wushu Changbing athletes, encompassing 16 discrete 3D kinetic metrics and five functional principal components (44). The findings provide valuable insights into the sex-specific force production mechanisms, providing a robust biomechanical foundation for coaches to design targeted, individualized training protocols, optimize key technical details, and potentially enhance athletic performance.

As a key component characterizing “Braking and Stabilization” during the terminal phase of the movement, PC3 demonstrated significant sexual dimorphism (*p* = 0.023). This not only reflects the adaptive technical strategies employed by females during the Changbing PiJi strike but also highlights their heightened demands for postural control. Drawing upon the research by (52) regarding proximal-to-distal momentum transfer, the high loading on PC3 suggests that that females may increase their neuromuscular control efforts during the terminal phase to better manage implement inertia and maintain bodily balance.

This mechanistic hypothesis is further supported by the discrete kinetic data in this study: although the total peak kinetic energy (T_full_peak_J) of females was significantly lower than that of males (*p* = 0.013), their lateral force variability [SD(X)] exhibited greater stability (males 0.485 vs. females 0.345, *p* < 0.001). This finding supports the perspective of (38) on the association between waveform characteristics and movement quality, suggesting that the higher PC3 scores in females likely represent an active control mechanism that optimizes the “signal-to-noise ratio” of the neuromuscular system, rather than merely passive braking. Under the physiological constraints of limited absolute strength, female athletes appear to minimize redundant kinetic noise to prioritize the structural integrity of the movement. Consequently, they achieve an efficient deceleration of the implement with lower energy expenditure, reflecting a highly adapted “low-power, high-stability” adaptive trait.

Compared to the “control-first” paradigm of females, the significant sex difference in PC4 (with males scoring significantly higher, *p* < 0.001) points toward a more propulsion-oriented kinetic drive strategy in males. This divergence is further elucidated by the directional specificity of the discrete impulse data: males exhibit significantly greater peak lateral force [Max(X), *p* = 0.012], whereas females demonstrate significantly higher total vertical impulse [Impulse(Z)] than males (*p* = 0.003). This finding offers a counterpoint to the traditional perspective that views vertical momentum merely as “kinetic dissipation,” suggesting that under conditions of limited explosive power, females appear to utilize increased vertical impulse to compensate for insufficient striking force (53, 54).

Conversely, males tend toward a “horizontal-propulsion-dominant” tendency [Impulse(Y), *p* = 0.058], aimed at efficiently translating ground reaction forces into horizontal acceleration. However, according to Signal-dependent Noise Theory, this pursuit of maximizing kinetic energy output is not without cost (55–57). The greater lateral force variability [SD(X), *p* < 0.001] observed in males likely reflects a biomechanical trade-off associated with sacrificing partial stability. This not only aligns with the “Stability-Propulsion Trade-off” mechanism proposed by (58), but also, as emphasized by (59), suggests that higher-order principal components like PC4 can effectively capture this dynamic micro-instability induced by the pursuit of maximal striking power.

Despite observed differences in energy output amplitude and kinetic control strategies between sexes, the 3D temporal structure of their force application remains highly conserved. The Generalized Motor Program (GMP) theory offers a valuable theoretical framework for understanding this phenomenon (60, 61). This study did not observe significant sex effects in the most explanatory components, namely PC1, PC2, and PC5 (cumulative variance explained: 53.34%, p>0.05), suggesting that these components likely represent the relatively stable “Fundamental Kinetic Modalities” within the Changbing PiJi technique (62). Temporal analysis of the discrete data further supports this observation: time to peak across all three spatial dimensions (Time_at_Max X, Y, Z) exhibited no significant sexual dimorphism, demonstrating a highly consistent temporal structure between sexes in core movement execution and gravity counteraction.

This result reveals the core spatiotemporal “Invariant Features” governed by task constraints and implement inertia, which remain relatively stable across changes in sex or muscle recruitment strategies (63). In other words, whether it is the aggressive “propulsion-dominant” pattern of males or the robust “control-first” strategy of females, the process of constructing the momentum transfer chain via vertical drive follows a highly consistent biomechanical template. Consequently, the ultimate discrepancy in athletic performance appears to be driven less by an alteration in the fundamental movement structure. Rather, it relies heavily on on the “Amplitude Scaling” of these core modalities-achieving diverse functional outputs by modulating the absolute magnitude of force within a shared spatiotemporal framework, instead of altering the core spatiotemporal structure itself.

## 5 Limitations

Although this study provided detailed insights into the sex-specific kinetic mechanisms of the PiJi technique in elite Wushu Changbing athletes, several limitations warrant consideration.

First, the non-contact shadow striking paradigm employed in this experiment lacks the inherent physical feedback mechanisms and reactive decision-making processes present in actual combat, which, to some extent, limits the ecological validity of the kinetic data (64). Second, all participants were right-dominant, potentially limiting the generalizability of the conclusions to left-handed populations. Finally, given that the sample consisted of elite athletes with long-term sport-specific adaptations, the spatiotemporal structures they likely represent a highly specialized adaptation tailored to a specific competitive level. Therefore, the generalizability of these findings to beginners or other age cohorts remains to be verified.

Future research would benefit from expanding the skill levels and demographic characteristics of the sample to comprehensively analyze the biomechanical evolution of this technique across different stages of skill acquisition.

## 6 Practical applications

This study reveals an inherent, cross-sex biomechanical architecture within the Changbing PiJi technique. Given that PC1, PC2, and PC5 constitute fundamental force production modalities with no significant sex differences, it is recommended to prioritize standardized training strategies during the foundational acquisition phase to construct normative core motor programs.

However, as competitive levels advance, the significant sex differences characterized by PC3 and PC4 suggest the potential benefits of transitioning away from traditional “mixed training” paradigms in favor of implementing sex-specific, differentiated interventions. For male athletes, future training studies might explore the theoretical utility of perturbation control training (65, 66) as a hypothesized means to manage lateral instability during maximal force exertion, thereby potentially reducing energy dissipation. Conversely, female athletes could explore horizontally-oriented resistance exercises (e.g., sled pushes) as a theoretical approach to enhance lower-limb explosive power and compensate for the relative deficit in horizontal propulsion, while simultaneously maintaining their terminal control advantages observed in PC3 (67).

It is noteworthy that the time to peak across all three spatial dimensions (X, Y, and Z axes) demonstrates a high degree of consistency between sexes. This comprehensive spatiotemporal uniformity suggests that the PiJi technique operates within a relatively stable kinetic framework. Consequently, it is recommended that training interventions align with this inherent 3D temporal rhythm-achieving the organic integration of physical conditioning and sport-specific technique without disrupting the movement's foundational spatiotemporal structure.

## 7 Conclusion

This study demonstrates that the PiJi technique among elite Wushu Changbing athletes exhibits cross-sex consistency in core force production rhythms, whereas sex differences are prominently manifested in the kinetic trade-off strategies between “propulsion” and “stability”. Males tend to adopt an aggressive “high-propulsion, low-stability” strategy to maximize impulse output, while females employ a “high-control, high-stability” braking strategy to optimize momentum transfer.These findings elucidate the physiological adaptation patterns of the PiJi technique within the traditional Chinese Shaolin staff system from a modern biomechanical perspective.

Consequently, these results provide an exploratory theoretical basis suggesting that differentiated, sex-specific training interventions might be beneficial in competitive training. For instance, future protocols could explore targeting the enhancement of stability control in males and power conversion capacity in females. However, given the cross-sectional nature of the current study, these practical inferences—including their potential efficacy in enhancing performance or mitigating injury risks—remain speculative. Future longitudinal and intervention-based studies are required to validate these clinical and practical applications. Furthermore, the analytical framework established herein provides a robust reference paradigm for investigating the biomechanical characteristics of other striking implement sports and exploring the sex-specific adaptation mechanisms of complex motor skills.

## Data Availability

The raw data supporting the conclusions of this article will be made available by the authors, without undue reservation.
